# Cross-border comparison of antibiotic prescriptions among children and adolescents between the north of the Netherlands and the north-west of Germany

**DOI:** 10.1186/s13756-016-0113-8

**Published:** 2016-04-18

**Authors:** Jan-Willem H. Dik, Bhanu Sinha, Alex W. Friedrich, Jerome R. Lo-Ten-Foe, Ron Hendrix, Robin Köck, Bert Bijker, Maarten J. Postma, Michael H. Freitag, Gerd Glaeske, Falk Hoffmann

**Affiliations:** Department of Medical Microbiology, University of Groningen, University Medical Center Groningen, Hanzeplein 1, 9713 GZ, Groningen, The Netherlands; Certe Laboratories for Infectious Diseases, Groningen, The Netherlands; Institute for Hospital Hygiene, University Medical Campus Oldenburg, Oldenburg, Germany; Institute of Medical Microbiology, University Hospital Münster, Münster, Germany; IADB, Department of Pharmacy, Unit of PharmacoEpidemiology & PharmacoEconomics, University of Groningen, Groningen, The Netherlands; Institute of Science in Healthy Aging & healthcaRE (SHARE), University of Groningen, University Medical Center Groningen, Groningen, The Netherlands; Department of Health Services Research, Carl von Ossietzky University Oldenburg, Oldenburg, Germany; Centre for Social Policy Research, Division Health Economics, Health Policy and Outcomes Research, University of Bremen, Bremen, Germany

**Keywords:** Antibiotics, Germany, Netherlands, Border research, Health services research

## Abstract

**Background:**

Antibiotic resistance is a worldwide problem and inappropriate prescriptions are a cause. Especially among children, prescriptions tend to be high. It is unclear how they differ in bordering regions. This study therefore examined the antibiotic prescription prevalence among children in primary care between northern Netherlands and north-west of Germany.

**Methods:**

Two datasets were used: The Dutch (IADB) comprises representative data of pharmacists in North Netherland and the German (BARMER GEK) includes nationwide health insurance data. Both were filtered using postal codes to define two comparable bordering regions with patients under 18 years for 2010.

**Results:**

The proportion of primary care patients receiving at least one antibiotic was lower in northern Netherlands (29.8 %; 95 % confidence interval [95 % CI]: 29.3–30.3), compared to north-west Germany (38.9 %; 95 % CI: 38.2–39.6). Within the respective countries, there were variations ranging from 27.0 to 44.1 % between different areas. Most profound was the difference in second-generation cephalosporins: for German children 25 % of the total prescriptions, while for Dutch children it was less than 0.1 %.

**Conclusions:**

This study is the first to compare outpatient antibiotic prescriptions among children in primary care practices in bordering regions of two countries. Large differences were seen within and between the countries, with overall higher prescription prevalence in Germany. Considering increasing cross-border healthcare, these comparisons are highly valuable and help act upon antibiotic resistance in the first line of care in an international approach.

## Background

Inappropriate use of antibiotics leads to significant clinical and economic problems due to resistant bacteria and increasingly limited anti-infective treatment options [1, 2]. The majority of antibiotics are prescribed in primary care, and children receive a large portion of these prescriptions [3, 4]. These primary care prescriptions of antibiotics are contributing to the world-wide resistance problem [5] and promotion of appropriate use is still of great importance [6]. Within the European Union there are large variations in outpatient antibiotic prescriptions. In the Netherlands, the level of prescriptions has traditionally been one of the lowest in Europe. In Germany, generally the prescription level is also relatively low [3]. However, when it comes to antibiotic prescriptions for children and adolescents, prescriptions in Germany seem to be higher than in other countries [7, 8]. Regarding antibiotic resistance Germany has also slightly higher levels than the Netherlands [9].

Due to a recent EU directive, patients can more easily obtain health services in other European member states. Directive 2011/24/EU creates the possibility for EU citizens to cross borders and seek healthcare in another country. This possibility for cross-border care is especially relevant for bordering regions such as the north of the Netherlands and north-west Germany, where Dutch healthcare centers can treat German patients and vice versa. Considering the potential effects of inappropriate antibiotic prescriptions in primary care, in particular antibiotic resistance development, it is relevant to look at differences in prescriptions between countries. It is therefore interesting to approach the topic of antimicrobial therapies and their possible unwanted effects by international cross-border collaboration, especially between bordering regions [10]. Notably, children and adolescents represent large group of recipients of antibiotics, in particular in outpatient care [3, 4]. The goal of this study was to examine the prevalence and most frequently prescribed antibiotics among children and adolescents in outpatient care in adjacent Dutch and German regions, and to compare this data. We also aimed to answer if different healthcare systems influence prescribing or if prescribing in regions gets more comparable the nearer they are to the border.

## Methods

### Study design and setting

**Fig. 1:**
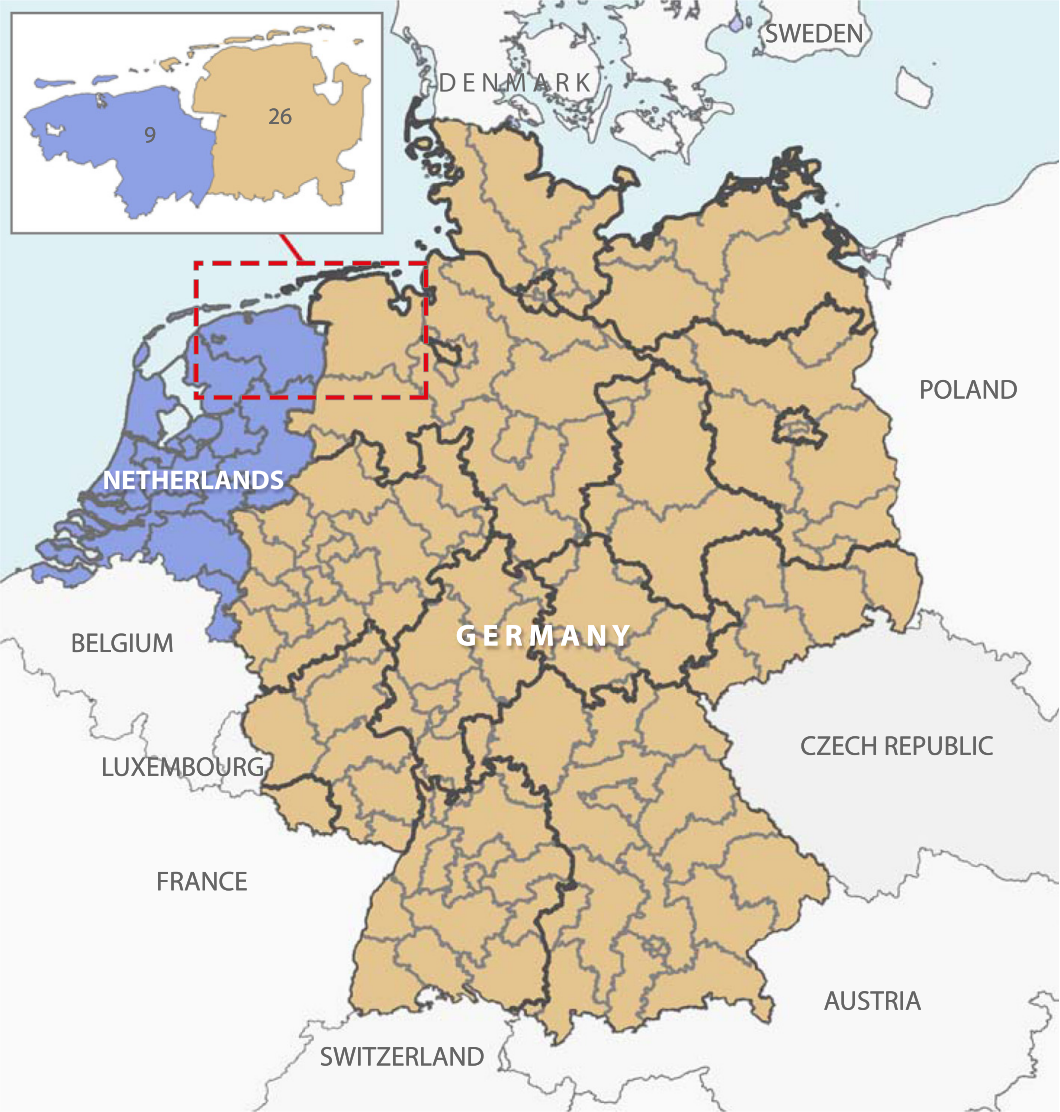
Study regions in north of the Netherlands (postal codes 9xxx; *n* = 36,747) and north-west Germany (postal codes 26xxx; *n* = 18,374)

To address the question, we chose a retrospective cross-sectional study in a predominantly rural region including analysis of outpatients’ data of a health insurance company and a pharmacy research database for north western Germany (postal codes 26xxx; part of Lower Saxony) and the northern Netherlands (postal codes 9xxx, Groningen/Drenthe). Both regions have no major geographic and infection risk differences, have about 1 million inhabitants and share a common green border (Fig. 1). Data comprises the year 2010 and we focused on persons aged 0 to 18 years. Orally administered antibiotics were selected based on the Anatomical Therapeutic Chemical (ATC) code J01 in the outpatient setting.

### Data sources

The German data including pharmacy dispensing data come from one of the largest health insurance companies, the BARMER GEK, representing approximately 8.4 million persons nationwide (of them about 104,000 in postal codes 26xxx). In total, there were 160 different statutory health insurance companies (including BARMER GEK) covering a total of 70 million persons (87 % of the German population) at the end of 2010, while the remaining inhabitants are privately insured. The study cohort consisted of persons who were insured at least one day in each of the four quarters of 2010. Due to this criterion, the vast majority of our study cohort has been continuously insured throughout the whole year but infants born in the first quarter could also be analyzed [11].

The Dutch data were derived from pharmacy dispensing data of the pharmacy research database (www.IADB.nl). The IADB comprises prescriptions derived from 55 community pharmacies in the northern part of the Netherlands and has in total approximately 600,000 persons in the database (of them about 263,000 in postal codes 9xxx). Since Dutch patients are in general registered at one single local pharmacy in their hometown, the chance of multiple prescriptions of one person being counted in different areas is relatively small. Registration is furthermore irrespective of healthcare insurance and age, gender and prescription rates among the database population have been representative for the whole of the Netherlands [12]. Prescription records are virtually complete due to the high patient-pharmacy commitment in the Netherlands, except for medication dispensed during hospitalization [12].

### Statistical analysis

Our main outcome was outpatient prescription prevalence, e.g. the proportion of children and adolescents receiving at least one prescription for a systemic antibiotic in 2010. Prevalence was stratified by sex, age groups (0–2, 3–6, 7–10, 11–13 and 14–17 years) and region of residence (six areas for the Netherlands and nine for Germany) alongside with 95 % confidence intervals (95 % CI). Furthermore, the most frequently prescribed antibiotic substances (by different ATC-codes) were studied. Statistical analyses were performed with SAS, Version 9.2 (SAS Institute Inc., Cary, NC). Maps were created with ESRI ArcGIS®, Version 10.2.2.

## Results

The study cohort consisted of 36,747 children and adolescents under the age of 18 years, living in the northern Netherlands (ranging between 649–20,739 individuals per region) and 18,374 from north-west Germany (ranging between 988–4,028 individuals per region). For the Netherlands, the distribution male vs. female was 50 % vs. 50 % and for Germany 51 % vs. 49 %.

**Fig. 2:**
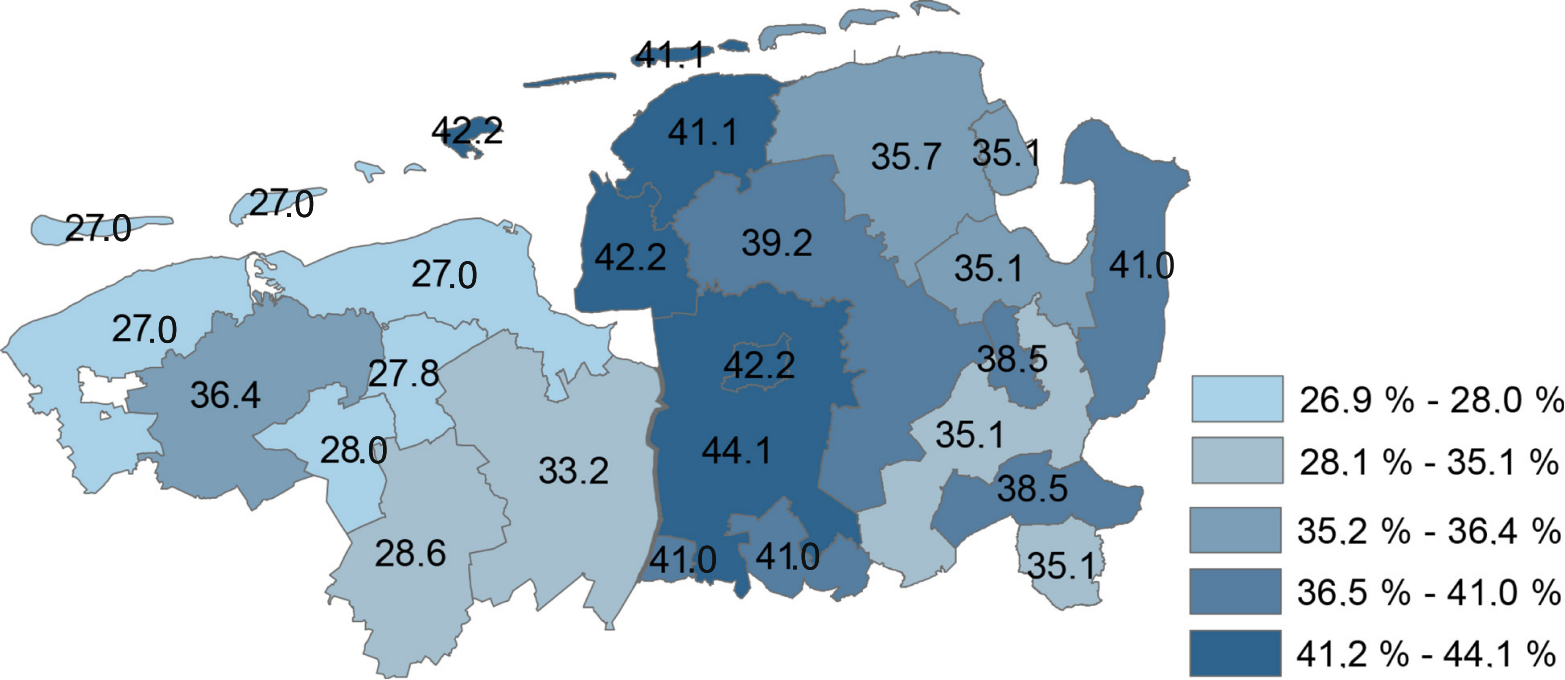
Small area variations (by postal codes) in the proportion of children and adolescents with prescriptions of antibiotics in the northern Netherlands and north-west Germany

Overall, the proportion of children and adolescents receiving at least one antibiotic course in 2010 was lower in the northern Netherlands (29.8 %; 95 % CI: 29.3–30.3) compared to north-west Germany (38.9 %; 95 % CI: 38.2–39.6). There were small area variations ranging from 27.0 to 36.4 % in the northern Netherlands and from 35.1 to 44.1 % in north-west Germany (Fig. 2). Prevalence stratified by region, sex and age groups is shown in Tables 1 and 2. The age groups with the highest proportion of prescriptions were children between 0–2 years (northern Netherlands vs. north-west Germany: 43.1 % vs. 49.9 %) and those between 3–6 years (37.4 % vs. 54.8 %). The proportion was considerably higher in Germany than in the Netherlands in all age groups, for males and for females. Males had a lower prevalence of antibiotic prescriptions than females for both Netherlands and Germany in all age groups, except for children aged 0–2 years old (Table 2).

**Table 1:** Small area variations (by postal codes) in the proportion of children and adolescents with prescriptions of antibiotics in the northern Netherlands and north-west Germany (with 95 % CI), by sex

Region/postal code (number of children included)	Males	Females	Total
Netherlands			
95, 96 (*n* = 10,439)	31.6 % (30.3–32.9)	34.7 % (33.4–36.0)	33.2 % (32.3–34.1)
90, 91, 99 (*n* = 649)	27.7 % (23.0–32.8)	26.1 % (21.3–31.4)	27.0 % (23.6–30.6)
92, 98 (*n* = 1,898)	36.2 % (33.2–39.4)	36.6 % (33.5–39.8)	36.4 % (34.2–38.6)
93 (*n* = 868)	28.0 % (23.9–32.5)	28.0 % (23.8–32.5)	28.0 % (25.0–31.1)
94 (*n* = 2,154)	28.0 % (25.3–30.8)	29.2 % (26.5–31.9)	28.6 % (26.7–30.6)
97 (*n* = 20,739)	26.7 % (25.8–27.6)	28.9 % (28.0–29.8)	27.8 % (27.2–28.4)
Germany			
261 (*n* = 4,028)	35.3 % (33.2–37.4)	34.9 % (32.8–37.0)	35.1 % (33.6–36.6)
262 (*n* = 988)	35.0 % (30.8–39.3)	42.1 % (37.6–46.6)	38.5 % (35.4–41.6)
263 (*n* = 1,744)	31.9 % (28.8–35.1)	38.4 % (35.2–41.7)	35.1 % (32.9–37.4)
264 (*n* = 1,959)	36.5 % (33.5–39.6)	34.9 % (31.8–37.9)	35.7 % (33.6–37.8)
265 (*n* = 1,102)	40.8 % (36.7–45.1)	41.4 % (37.2–45.6)	41.1 % (38.2–44.1)
266 (*n* = 2,476)	38.5 % (35.8–41.2)	40.0 % (37.2–42.8)	39.2 % (37.3–41.2)
267 (*n* = 1,263)	40.7 % (36.7–44.7)	43.6 % (39.8–47.5)	42.2 % (39.5–45.0)
268 (*n* = 3,642)	42.0 % (39.8–44.3)	46.2 % (43.9–48.6)	44.1 % (42.5–45.7)
269 (*n* = 1,172)	41.1 % (37.2–45.1)	40.8 % (36.7–45.0)	41.0 % (38.1–43.8)

**Table 2:** Proportion of children and adolescents with prescriptions of antibiotics in the northern Netherlands and north-west Germany (with 95 % CI), by sex and age group

	Males		Females		Total	
Age	Netherlands (*n* = 18,229)	Germany (*n* = 9,283)	Netherlands (*n* = 18,518)	Germany (*n* = 9,091)	Netherlands (*n* = 36,747)	Germany (*n* = 18,374)
0–2 years. (*n* = 8,075 resp. *n* = 1,770)	44.5 % (43.0–46.0)	53.1 % (49.8–56.4)	41.5 % (39.9–43.1)	46.6 % (43.2–50.0)	43.1 % (42.0–44.2)	49.9 % (47.6–52.3)
3–6 years. (*n* = 7,464 resp. *n*∓3,488)	35.2 % (33.8–36.8)	56.2 % (53.9–58.5)	39.9 % (38.2–41.5)	53.3 % (50.9–55.7)	37.4 % (36.3–38.5)	54.8 % (53.2–56.5)
7–10 year. (*n* = 6,916 resp. *n* = 4,292)	19.6 % (18.3–20.9)	35.4 % (33.3–37.4)	27.7 % (26.1–29.2)	37.4 % (35.4–39.5)	23.4 % (22.4–24.4)	36.4 % (35.0–37.9)
11–13 years. (*n* = 5,078 resp. *n* = 3,733)	16.3 % (14.9–17.8)	27.7 % (25.6–29.8)	19.6 % (18.0–21.2)	28.9 % (26.9–31.0)	17.9 % (16.8–18.9)	28.3 % (26.9–29.8)
14–17 years. (*n* = 9,214 resp. *n* = 5,091)	20.7 % (19.4–22.1)	29.1 % (27.4–30.9)	25.2 % (24.0–26.3)	39.1 % (37.2–41.0)	23.4 % (22.6–24.3)	34.0 % (32.7–35.3)
Total (*n* = 36,747 resp. *n* = 18,374)	28.7 % (28.0–29.4)	37.9 % (36.9–38.9)	30.9 % (30.3–31.6)	39.9 % (38.9–40.9)	29.8 % (29.3–30.3)	38.9 % (38.2–39.6)

Distributions of the antibiotic substances varied between the two bordering regions. Amoxicillin was the most frequently prescribed substance in both regions, 49.6 % of all prescriptions in the Netherlands versus 21.1 % in Germany. Another profound difference was found for second generation cephalosporins, which in the Netherlands is reserved as a second line antibiotic. These antibiotics comprised 25 % of the prescriptions in Germany and less than 0.1 % in the Netherlands.

The five most frequent prescribed antibiotics for all paediatric age groups covered 81.0 % of the total number of prescriptions in the Netherlands vs. 64.3 % in Germany (Table 3). In both countries, the percentage of top 5 prescriptions decreased with age: in the Netherlands from 94.2 % in 0–2 year olds to 69.0 % in 14–17 year olds and in Germany from 76.4 % in 0–2 year olds to 53.6 % in 14–17 year olds.

**Table 3:** The top 5 most prescribed antibiotic substances in the northern Netherlands and north-west Germany, by age group and the total

Age	Netherlands	%	Germany	%
0–2 years.				
1st	Amoxicillin	70.7 %	Cefaclor	29.5 %
2nd	Amoxicillin-clavulanate	10.5 %	Amoxicillin	22.0 %
3rd	Clarithromycin	6.6 %	Erythromycin	14.2 %
4th	Sulfamethoxazole and trimethoprim	3.7 %	Cefuroxime	5.9 %
5th	Pheneticillin	2.7 %	Phenoxymethylpenicillin	4.8 %
Total of the 5 most prescribed		94.2 %		76.4 %
3–6 years.				
1st	Amoxicillin	56.1 %	Amoxicillin	22.2 %
2nd	Amoxicillin-clavulanate	13.6 %	Cefaclor	21.9 %
3rd	Clarithromycin	8.9 %	Phenoxymethylpenicillin	10.5 %
4th	Pheneticillin	4.5 %	Erythromycin	10.0 %
5th	Sulfamethoxazole and trimethoprim	4.2 %	Sulfamethoxazole and trimethoprim	7.9 %
Total of the 5 most prescribed		87.4 %		72.6 %
7–10 year.				
1st	Amoxicillin	43.5 %	Amoxicillin	19.7 %
2nd	Amoxicillin-clavulanate	13.5 %	Cefaclor	18.1 %
3rd	Clarithromycin	9.6 %	Erythromycin	11.2 %
4th	Nitrofurantoin	7.8 %	Phenoxymethylpenicillin	11.1 %
5th	Flucloxacillin	6.6 %	Sulfamethoxazole and trimethoprim	8.1 %
Total of the 5 most prescribed		81.1 %		68.2 %
11–13 years.				
1st	Amoxicillin	36.5 %	Amoxicillin	22.4 %
2nd	Amoxicillin-clavulanate	13.2 %	Cefaclor	12.6 %
3rd	Clarithromycin	10.0 %	Cefuroxime	10.7 %
4th	Nitrofurantoin	8.9 %	Sulfamethoxazole and trimethoprim	8.7 %
5th	Sulfamethoxazole and trimethoprim	5.8 %	Phenoxymethylpenicillin	8.1 %
Total of the 5 most prescribed		74.4 %		62.5 %
14–17 years.				
1st	Nitrofurantoin	19.7 %	Amoxicillin	19.7 %
2nd	Amoxicillin	16.0 %	Cefuroxime	9.4 %
3rd	Doxycycline	14.8 %	Azithromycin	8.4 %
4th	Amoxicillin-clavulanate	10.5 %	Phenoxymethylpenicillin	8.1 %
5th	Pheneticillin	8.1 %	Sulfamethoxazole and trimethoprim	8.0 %
Total of the 5 most prescribed		69.0 %		53.6 %
0–17 years.				
1st	Amoxicillin	49.6 %	Amoxicillin	21.1 %
2nd	Amoxicillin-clavulanate	11.9 %	Cefaclor	17.3 %
3rd	Clarithromycin	7.7 %	Phenoxymethylpenicillin	9.0 %
4th	Nitrofurantoin	7.0 %	Erythromycin	9.0 %
5th	Pheneticillin	4.8 %	Cefuroxime	7.9 %
Total of the 5 most prescribed		81.0 %		64.3 %

## Discussion

### Study findings and implications

This is one of the first studies to look at outpatient antibiotic prescriptions among children in the bordering regions of two countries. Considering the importance of appropriate antibiotic use, the ability for patients to seek healthcare services abroad, and the large differences between healthcare systems and guidelines, a cross-border comparison is of great interest. Furthermore, these comparisons may be beneficial to inform healthcare providers and to discuss best clinical practice. We observed considerable differences between the two countries. This may indicate that improvements can be achieved. Mainly on the distribution of substances, differences were profound: second generation cephalosporins (i.e., mostly oral cefuroxime) were prescribed in 25 % of the cases for the German patients, while almost none of the Dutch children received this type (<0.1 %). Given the low rate of oral bioavailability and the high selective pressure due to these substances, they are avoided wherever possible in ambulatory paediatric care in the Netherlands. It would be highly interesting and relevant to perform further research into the reasons for prescribing second generation cephalosporins in Germany and its long term effects.

These differences in prescriptions of antibiotics for outpatients between European countries have been reported earlier, although not for bordering regions. There are however comparisons showing differences between Germany and the Netherlands in general [3], as well as for children [9]. In the Netherlands, guideline adherence is higher compared to Germany [13], which might also be a reason for different distributions of substances between both countries. In 7–17 years old children, nitrofurantoin was among the top 5 antibiotics in the Netherlands but not in Germany. In Germany, there may still be hesitance to prescribe nitrofurantoin due to a history of warnings in the past (pulmonary fibrosis, neuropathy and liver damage) [14].

In addition, the occurrence of resistant bacteria such as MRSA differs also significantly between the bordering regions of the Netherlands and Germany, with up to 32-fold higher MRSA incidence in the German border region compared to the adjacent Dutch border region [15]. In addition, higher resistance rates were also observed for classical community-acquired pathogens such as pneumococci where penicillin-resistance involved 1.9 % of invasive isolates in Germany vs. 0.2 % in the Netherlands in 2013 [16]. The Dutch prevalence data observed in this study are comparable to earlier studies, indicating a quite stable use and also corroborating the fact that the IADB database can be considered as representative for the whole county [9, 17]. Comparing various German studies there is a bit more variation, but it is known that there is a quite large regional variation of outpatient antibiotic prescriptions within the country. The north-western part of Germany, which is included in this study, is one of the higher prescribing regions [18, 19].

For both datasets, clinical indications for the prescriptions analyzed were not known. For the Netherlands, a large survey showed that the primary diagnosis for children coming to general practitioners is lower respiratory tract infections [20]. Antibiotic prescriptions by general practitioners for children in the Netherlands are mainly for acute otitis media and bronchitis, and especially broad-spectrum antibiotics are still prescribed inappropriately [21]. Dutch guidelines regarding antibiotic treatment are very strict. Otitis media guidelines recommend antibiotics only when there are other risk factors for complications or with severe general symptoms [22]. For respiratory tract infections, antibiotics are only recommended in the case of pneumonia [23]. In Germany most diagnoses for children (0–15 years) in an outpatient setting were upper respiratory tract infections without a focus, fever without a focus and acute bronchitis [24]. Antibiotic prescriptions for this group are mainly given for acute tonsillitis, bronchitis and otitis media, for all of which appropriateness of antibiotics is debatable [8]. The German guideline for otitis media recommends antibiotic treatment only to be started after two days, thereby being less conservative than the Dutch counterpart [25]. The general guideline for bronchitis states that when uncomplicated, antibiotics are not recommended and should be avoided [26].

Influence of parents on the prescribed antibiotic treatment seems to be relatively small. A European survey, although not performed in the Netherlands or Germany, showed that patients tend to adhere to the decision of the general practitioner even when they disagree [27]. When they disagree, they have a tendency to be more conservative than the physician [28]. A survey in Germany confirms this and shows that the large majority of patients understand the limitations of antibiotic treatment for indications like the common cold [29]. The influence of the family practitioner or paediatrician thus seems to be often underestimated, whereas they show indeed a high inter-individual variation in their prescription pattern [30]. Perceptions of antibiotic resistance among general practitioners also differs between countries [31], probably also leading to different prescription behaviour. A combination of these aspects is most likely leading to the differences between the Netherlands and Germany.

### Strengths and limitations

The major strength of this study is the unique dataset of two bordering regions coming from countries with different healthcare systems and antibiotic prescribing policies. Other studies compared nationwide data (either from a subset of databases or up to 100 % coverage such as most of ESAC). However, as shown here for the first time, there are also large small area variations among and between bordering regions from two different countries. Studies comparing national consumption data, aggregate these data and variations within a country are then lost, making it impossible to effectively compare bordering regions. We were able to include about 37,000 children and adolescents living in the northern Netherlands as well as 18,000 living in north-west Germany. However, especially in the Netherlands our cohort size differs relevantly per area (depending on the distribution of pharmacies included in the IADB.nl database). Therefore, in some areas several postal codes were combined. Age distribution and drug utilization of the patients included in the database is, however, representative for the total Dutch population [12]. For Germany, the sample size was somewhat smaller than for the Netherlands. German data were derived from a large health insurance fund and we know that differences exist regarding socioeconomic status and morbidity between these insurance funds [32]. Such differences were found in children and adolescents, too, but the utilization of medications within the specific fund we used was quite comparable to the complete German population [33]. Unfortunately, we had no access to diagnoses and indications for which antibiotics were prescribed. These data would be relevant in determining the appropriateness of the (antibiotic) treatments, and also could shed light on the question if some patients might even be undertreated. It seems, that coming closer to the border increases antibiotic consumption in both countries. One explanation might be, that these parts of the country are furthest away from an academic centre. These (rural) parts of the countries are also socio-economically weaker compared to the more densely populated parts. Such a lower socio-economic status appears to influence antibiotic prescribing, although it is unclear to which extent [34, 35]. However, more precise information is not available within the datasets used. This study should form a starting point for (regional) antimicrobial stewardship programs focusing on general practitioners and outpatients. This group is still somewhat neglected in stewardship programs, but these data show that there is a lot to gain.

It is important to keep in mind that the structures and organization of the two healthcare systems differ substantially between the Netherlands and Germany. In the former, there are only family practitioners in private practice, whereas nearly all specialists are working in outpatient clinics in (larger) hospitals. Hence, the data analyzed in this study primarily contain prescription data for these family physicians. In Germany, the majority of medical specialists, including paediatricians, is working in private practice (or consortiums). The German data set comprised also the data from these specialists. Previous analysis for the whole of Germany showed that 49 % of the prescriptions came from paediatricians and 35 % from general practitioners [19]. This may also influence individual prescribing patterns due to a variety of reasons and cause a bias due to more severe cases treated by specialists on the German side of the border, although we hypothesize that severe cases are most likely send to a hospital and are thus not included in this dataset. One may speculate that the more individualized healthcare system for primary care in Germany might lead to a more heterogeneous healthcare behaviour than the more peer group-dependent gatekeeper system in the Netherlands. Prescribing patterns are influenced by many different factors. Other differences between the Netherlands and Germany (e.g. medical education, performing microbiological diagnostics or healthcare insurance system) are most likely also of influence, however to which degree is uncertain and should be subject to further investigation.

## Conclusions

Concluding, this study shows clear differences between primary care antibiotic prescriptions for children and adolescents in the bordering regions of the Netherlands and Germany. Especially in the age group of 3–6 year-old children, prescriptions are more frequent in Germany. Overall there also seems to be a tendency to prescribe broader substances in Germany compared to the Netherlands. An evaluation like this is especially interesting, considering that most antibiotics in a primary care setting are prescribed for children. Keeping in mind the effects that sub-optimal antibiotic treatments can have, these comparisons of bordering regions between two countries provide an opportunity to learn from each other and collaborate internationally in order to counteract the problems of rising antibiotic resistance from a first line of care perspective.

## References

[1] Goossens H (2009). Antibiotic consumption and link to resistance. Clin Microbiol Infect.

[2] Cosgrove S (2006). The relationship between antimicrobial resistance and patient outcomes: mortality, length of hospital stay, and health care costs. Clin Microbiol Infect.

[3] European Centre for Disease Prevention and Control (2014). Surveillance of antimicrobial consumption in Europe 2012.

[4] Brauer R, Ruigómez A, Downey G, Bate A, Garcia Rodriguez LA, Huerta C (2015). Prevalence of antibiotic use: a comparison across various European health care data sources. Pharmacoepidemiol Drug Saf.

[5] Costelloe C, Metcalfe C, Lovering A, Mant D, Hay AD (2010). Effect of antibiotic prescribing in primary care on antimicrobial resistance in individual patients: systematic review and meta-analysis. BMJ.

[6] Earnshaw S, Mancarella G, Mendez A, Todorova B, Magiorakos AP, Possenti E (2014). European Antibiotic Awareness Day: a five-year perspective of Europe-wide actions to promote prudent use of antibiotics. Euro Surveill.

[7] Holstiege J, Garbe E (2013). Systemic antibiotic use among children and adolescents in Germany: a population-based study. Eur J Pediatr.

[8] Holstiege J, Schink T, Molokhia M, Mazzaglia G, Innocenti F, Oteri A (2014). Systemic antibiotic prescribing to paediatric outpatients in 5 European countries: a population-based cohort study. BMC Pediatr.

[9] European Centre for Disease Prevention and Control. Antimicrobial resistance surveillance in Europe 2012. In: Annual Report of the European Antimicrobial Resistance Surveillance Network (EARS-Net). Stockholm, Sweden: ECDC. 2013. Report No.: TQ-AM-13-001-EN-C.

[10] Friedrich AW, Daniels-Haardt I, Köck R, Verhoeven F, Mellmann A, Harmsen D (2008). EUREGIO MRSA-net Twente/Münsterland--a Dutch-German cross-border network for the prevention and control of infections caused by methicillin-resistant Staphylococcus aureus. Euro Surveill.

[11] Hoffmann F, Petermann F, Glaeske G, Bachmann CJ (2012). Prevalence and comorbidities of adolescent depression in Germany. An analysis of Health Insurance Data. Z Kinder Jugendpsychiatr Psychother.

[12] Visser S, Schuiling-Veninga CCM, Bos JHJ, de Jong-van den Berg LTW, Postma MJ (2013). The population-based prescription database IADB.nl: its development, usefulness in outcomes research and challenges. Expert Rev Pharmacoecon Outcomes Res.

[13] Philips H, Huibers L, Hansen EH, Christensen MB, Leutgeb R, Klemenc-Ketis Z (2014). Guidelines adherence to lower urinary tract infection treatment in out-of-hours primary care in European countries. Qual Prim Care.

[14] Adverse Effects: Urinary tract chemotherapeutic Nitrofuratoin – Organ damaging and outdated. Arznei Telegramm. 1993;11:128

[15] van Cleef BAGL, Kluytmans JAJW, van Benthem BHB, Haenen A, Monen J, Daniels-Haardt I (2012). Cross border comparison of MRSA bacteraemia between The Netherlands and North Rhine-Westphalia (Germany): a cross-sectional study. PLoS ONE.

[16] ECDC. Antimicrobial resistance interactive database (EARS-Net). ECDC. 2015. http://ecdc.europa.eu/en/healthtopics/antimicrobial_resistance/database/Pages/database.aspx. Accessed 15 July 2015

[17] de Jong J, van den Berg PB, de Vries TW, de Jong-van den Berg LTW (2008). Antibiotic drug use of children in the Netherlands from 1999 till 2005. Eur J Clin Pharmacol.

[18] Kern WV, de With K, Nink K, Steib Bauert M, Schröder H (2006). Regional variation in outpatient antibiotic prescribing in Germany. Infection.

[19] Koller D, Hoffmann F, Maier W, Tholen K, Windt R, Glaeske G (2013). Variation in antibiotic prescriptions: is area deprivation an explanation? Analysis of 1.2 million children in Germany. Infection.

[20] van der Linden MW, van Suijlekom-Smit LWA, Schellevis FG, van der Wouden JC. [Second nationwide study on diseases and procedures at general practioners]. Utrecht, the Netherlands: NIVEL. 2005

[21] Otters HBM, van der Wouden JC, Schellevis F, van Suijlekom-Smit LWA, Koes B (2004). Trends in prescribing antibiotics for children in Dutch general practice. J Antimicrob Chemother.

[22] NHG. [Acute paediatric otitis media]. NHG. 2014. https://www.nhg.org/standaarden/samenvatting/otitis-media-acuta-bij-kinderen. Accessed 13 May 2015

[23] NHG. [Acute coughing]. NHG. 2013. https://www.nhg.org/standaarden/samenvatting/acuut-hoesten. Accessed 13 May 2015

[24] Grobe TG, Dörning H, Schwartz F. [BARMER GEK Physician's report 2011]. Berlin, Germany: Asgard-Verlag. 2012

[25] DEGAM. [Otitis media]. DEGAM. 2014. http://www.degam.de/files/Inhalte/Leitlinien-Inhalte/Dokumente/DEGAM-S3-Leitlinien/LL-07_Kurzversion_Ohrenschmerzen_AWMF%20053_009.pdf. Accessed 13 May 2015

[26] DEGAM. [Acute coughing]. DEGAM. 2014. http://www.degam.de/files/Inhalte/Leitlinien-Inhalte/Dokumente/DEGAM-S3-Leitlinien/Kurzversion_Akuter%20Husten_20140320.pdf. Accessed 13 May 2015

[27] Brookes-Howell L, Wood F, Verheij T, Prout H, Cooper L, Hood K (2014). Trust, openness and continuity of care influence acceptance of antibiotics for children with respiratory tract infections: a four country qualitative study. Fam Pract.

[28] Hawkings NJ, Butler CC, Wood F (2008). Antibiotics in the community: a typology of user behaviours. Patient Educ Couns.

[29] Faber MS, Heckenbach K, Velasco E, Eckmanns T (2010). Antibiotics for the common cold: expectations of Germany’s general population. Euro Surveill.

[30] Cars H, Håkansson A (1997). Prescriptions of antibiotics for children. Prescribing habits of district, hospital, and private physicians. Scand J Prim Health Care.

[31] Wood F, Phillips C, Brookes Howell L (2013). Primary care clinicians’ perceptions of antibiotic resistance: a multi-country qualitative interview study. J Antimicrob Chemother.

[32] Hoffmann F, Icks A (2012). Structural differences between health insurance funds and their impact on health services research: results from the Bertelsmann Health-Care Monitor. Gesundheitswesen.

[33] Hoffmann F, Bachmann CJ (2014). Differences in sociodemographic characteristics, health, and health service use of children and adolescents according to their health insurance funds. Bundesgesundheitsbl Gesundheitsforsch Gesundheitsschutz.

[34] Covvey JR, Johnson BF, Elliott V, Malcolm W, Mullen AB (2014). An association between socioeconomic deprivation and primary care antibiotic prescribing in Scotland. J Antimicrob Chemother.

[35] Ternhag A, Grünewald M, Nauclér P, Wisell KT (2014). Antibiotic consumption in relation to socio-demographic factors, co-morbidity, and accessibility of primary health care. Scand J Infect Dis.

